# Mentalities and Mind-Sets: The Skeleton of Relative Stability in Psychology’s Closet

**DOI:** 10.5964/ejop.v15i3.2103

**Published:** 2019-09-27

**Authors:** Gordon Sammut

**Affiliations:** a University of Malta, Malta; Webster University Geneva, Switzerland

Over its brief history, the discipline of psychology has seen its fair share of crises (Farr, 1991). In the grand scheme of academic scholarship, it has sandwiched itself between the natural and the social sciences. This positioning, in itself, has fuelled fierce as well as productive debates concerning the discipline’s scope, its phenomenology, epistemology, and methodology (see Toomela & Valsiner, 2010). The latest crisis to afflict the discipline is the infamous ‘replication crisis’, precipitated by the Reproducibility Project which claimed that it was only able to find evidence for successful replication of published psychological findings in 47% of cases (i.e. less than chance, or 50%).

No doubt, many expected a higher replication rate, betraying a long held but little researched assumption concerning the immutability of psychological phenomena. To put it simply, many expect psychological findings to replicate invariably across diverse sociocultural contexts in the same way that, for instance, medical diagnoses do (see Sammut, Foster, Salvatore, & Andrisano-Ruggieri, 2016). If one is diagnosed with diabetes in the US, one does not expect different insulin uptake upon migration to China. Similar expectations are levelled at psychology, based chiefly upon the discipline’s claims to knowledge concerning psychopathology and personality. Like medical research, psychological scholarship here serves to typify and characterize individuals in ways that facilitate treatment of psychological symptoms, conditions, or disorders. Unlike medical research, however, psychological functioning seems to be amenable to, if not contingent upon, sociocultural influences. For instance, we are not entirely sure whether the human personality is primarily composed of the Big Five trait constellation (Costa & McCrae, 1992), or whether it becomes Seven if one is socialised in China (Wang, Cui, & Zhou, 2005; Zhou, Saucier, Gao, & Liu, 2009).

A recent commentary by Baucal, Gillespie, Krstić, & Zittoun (2019) argues that the discipline has, over the years, entertained three basic assumptions concerning the universality of psychological phenomena and the extent to which these demonstrate variable manifestation in diverse ecological contexts (Gigerenzer, 2000). The results emerging from the Reproducibility Project have thrown into crisis research programmes resting on assumptions of immutability. Conversely, they have provided support to research traditions that have assumed otherwise. In this contribution, I propose that the challenge precipitated by the replication crisis may have its silver lining, inasmuch as it requires the discipline to confront a skeleton in its closet – the issue of *relative stability*. In so doing, I query whether there might be something that psychologists might have missed along the way. Specifically, I propose that psychological research might be converging on a range of phenomena that lie at the heart of the dynamic stability that has long afflicted personality research (see Connelly, Ones, & Chernyshenko, 2014), but that might help explain how humans with genetically-rooted dispositions are capable of adapting to situated circumstances (Gigerenzer, 2000; Sammut, Foster, Salvatore, & Andrisano-Ruggieri, 2016.

## The Problem of People Who Kill Themselves and a Lot of Others With Them

‘Charlie Hebdo’, ‘9/11’, ‘the London bombings’ – these terms today need no contextualisation; they are remembered as terrorist events by virtue of their virality. The issue of individual differences in the endorsement of militant extremist beliefs has been, for some time now, a matter of intensive scientific research (Sageman, 2004). The overarching aspiration in this line of inquiry is to understand the root cause of extremist violence. A promising line of inquiry is the investigation of the *extremist mindset*. Međedović & Knežević (2019) claim psychopathology inspired research amongst militants and terrorists has yielded mixed empirical results. The issue has been noted since Arendt’s (1963) landmark analysis of Eichmann’s trial in Jerusalem, convicted and executed for war crimes perpetrated during his tenure as a Nazi officer. According to Arendt, Eichmann was no deranged psychopath, but “terribly and terrifyingly normal” (p. 276).

Međedović & Knežević (2019) investigated the role played by certain personality traits in an effort to identify a constellation marking the militant extremist personality (p. 92). The trait constellation in question is Disintegration, defined as “a proneness to see and feel connections among factually unrelated phenomena” (p. 93). This is investigated in light of the Dark Tetrad of human personality (Paulhus, 2014). The study is moot on the point as to whether this proneness is an individual psychological vulnerability associated with personality structures of a certain type, or whether this is an ideological vulnerability associated with ideologies of a certain type. That is, is extremism a function of the personality structure of the individual, or a function of the radical component in organized beliefs?

The authors report that the Militant Extremist Mindset [MEM] is predicted by a subtle balance of dispositions (p. 101), although sadism is demonstrated to be unrelated to psychopathy, which contradicts previous findings. The crucial issue, however, is whether MEM transpires similarly in clinical populations as subclinical ones, that is whether individual differences transpire in degree or in type. If the difference is in degree, then it is fair to investigate clinical traits in subclinical populations focusing on trait-like manifestations, as per the cited inquiry. But if the difference is in type, then the findings concerning one population do not generalise to the other and the expectation of replicating findings across this contextual diversity is misguided. Crucially, in the present case, the authors proffer a situational explanation for militant extremism that is neither construed in terms of a characteristic trait constellation marking the extremist personality type, nor in substantive ideological terms typifying certain beliefs as radical or extremist. Međedović & Knežević (2019) go on to claim that “In a context where everyone is using others for their own purposes, it becomes justifiable to employ the same strategies in order to change the state of affairs” (p. 100). In other words, in certain situations militant terrorism, terrifying as it may be, is justifiably normal.

This construal, however, strikes at the heart of personality research based on the individual differences tradition that also aspires to characterise types. If the predictive value of personality dispositions is contingent on their activation in specific situations, then their predictive validity, along with the expectation of replicability, is necessarily limited due to situational contingencies that are in themselves wholly unpredictable. To take stock, personality research has, broadly speaking, discerned trait-like structures that are seemingly associated with determined inclinations. The fruition of these inclinations in behaviour, however, is contingent on situational circumstances that are in themselves, open-ended. That is, if the stability of personality structures is only discernible in the specific situation, that is, if stability is relative – not to cultural context, but to situational context – then the burden facing personality researchers to map the precise structure of situational influences acting on the agent at the particular moment in which the structure has evinced becomes, truly, an untenable task. Along with the expectation of trans-situational replicability. Clearly, it is time for the discipline to acknowledge that universalistic aspirations regarding human cognition and personality have not been supported and rather than fixing statistics with more statistics (Baucal, Gillespie, Krstić, & Zittoun, 2019), it is time for the discipline to consider more deeply the range of phenomena that enable human adaptation to *situational* contingencies, given some stability in both personality dispositions and sociocultural contexts.

## Situated Cognition

A critical concern that has underlined numerous other debates in psychology is whether knowledge of psychological structures and processes serves descriptive or predictive purposes. For instance, does knowledge of the existence of racial stereotypes in a population serve to (aside from describing the state of the distribution of racial stereotypes in the specific population at the specific point in time it is investigated) predict correlated outcomes, such as frequency of hate crimes, on the basis of knowledge of the relations between racial stereotypes and hate crimes *observed in other instances*? The extension from descriptive research to inferential research aspiring to describe underlying and invariant processes has been long challenged theoretically (Harré & Secord, 1972) and, if the findings of the Reproducibility Project are anything to go by, now also empirically (Open Science Collaboration, 2015).

A situated view of personality and cognition counterbalances both foci. As Smith & Semin (2007) have argued, the situated view proposes that any description of human behaviour is only meaningful if it serves to establish grounds for communicative mutuality. This is due to the fact that human cognition is motivated (Kunda, 1990) and predisposed towards sociality (Tomasello, Carpenter, Call, Behne, & Moll, 2005). Human cognition enables individuals to form social identities around collective interests (Sammut, 2012). This form of sociality is only relatively enduring – as individuals’ interests change, the composition of the social group ebbs and flows, resulting in situational influences on group members. The situated view opposes the idea that inclinations flow invariably from autonomous, invariant cognitive processes located within independent agents. It proposes instead that motivated functions determine the nature of these processes.

For instance, an agent’s emotional state influences behavioural outcomes in the situation aside from sociocultural features of the society in which the behaviour occurs, or the personality dispositions of the agent involved. The situated emotional state of the agent is unstable in ways that go beyond general psychological orientations (such as personality structure) and are contingent on the unpredictable. For instance, need for closure research has demonstrated that time pressure (e.g. running late) leads to cognitive shortcuts in decision-making (Kruglanski, 2004). This suggests that if certain behavioural responses do not serve a motivated function, the cognitive processes leading to them will not be activated (Smith & Semin, 2007, p. 133). Consequently, this invites researchers to specify not only the psychological processes observed in themselves, but more importantly, the boundaries limiting their activation. The variable potential that inheres in personality structures, making stability in behavioural influences as a function of trait dispositions merely *relative*, provides plenty of scope for inquiry in its own right. I propose this might be key to understanding, more deeply, the human potential for situational adaptation.

## The Explanation of Social Behaviour

Psychological researchers investigating stable dispositional orientations in human personality have reliably observed, over the years, that personality structures are inherently unstable across variable circumstances. Specifically, it seems that the factor concerned with ‘Openness to Experience’ might have something to do with reconfigurations in the personality structure (Connelly, Ones, & Chernyshenko, 2014). I argue that such reconfigurations are adaptive and contingent on the individual’s situated circumstances. I propose that personality structure resembles a psychological camouflage by which dispositional inclinations are reconfigured (i.e. the personality structure is re-structured) to match situated ecological demands.

As noted, personality researchers looking for stable structures have routinely observed instability in personality dynamics. Conversely, researchers investigating social-psychological dynamics have routinely observed stable structures. By analogy to the Rubin Vase, those looking for a chalice have observed human faces whilst those searching for human faces have observed a chalice. Rather than an empirical inconsistency, I argue that this is a case of convergence in disparate research traditions.

The Big Five model of human personality comprised of a five-factor personality structure is very well-known (Costa & McCrae, 1992). The premise underlying this personality model is that human behaviour is genetically-based, at least in part, through inherent dispositions that develop in individuals over the course of maturation. By the time they reach adulthood, human beings develop an idiosyncratic personality, rooted in a constellation of traits shaped by genetic influences, that predisposes them to characteristic behavioural responses. What this model falls short in explaining is how and why these trait-based dispositions may change in the same individual over time, or why behavioural responses shaped by the same trait dispositions are inconsistent across situations for the same individuals. An equally well-known model that is similarly based on factor analytic techniques is the Cultural Dimensions model (Hofstede, 1991). The premise underlying this sociological model is that human behaviour is a function of cultural values transmitted through socialisation. Different cultures prioritise different values, depending on which cultural dimensions are salient in different societies, that lead to individuals expressing this cross-cultural variability in behavioural outcomes. What this model falls short in explaining is how and why some individuals demonstrate similar behavioural dispositions across different cultures, or how within a given culture individual differences may be highly marked. These two models are amongst similar efforts that have aimed to map out psychological and sociological structures in an effort to account for behavioural diversity. Both programmes, however, are prone to *relative* influences that lead to observed structural changes over time, situations, people, and contexts.

On the other hand, three other research programmes have investigated belief dymanics. The symbolic universes programme has investigated dynamics of sense-making in Europe in response to the financial and immigration crises. The social axioms programme sought to investigate cross-cultural differences in core beliefs that can account for behavioural diversity within and across cultures. The moral frameworks programme has studied the fundamental moral choices individuals make when they opt for a particular behavioural response that is justified in a given context against similarly justifiable alternative responses that lie at the individual’s disposal. All three programmes have claimed that behaviour is responsive to contextual characteristics that may precipitate changes in the current order. All three have also reported five loosely *stable* structures that seem to play a role in how individuals respond to other people and the events happening around them.

### Symbolic Universes

Salvatore et al. (2018) propose that human cognition is organised around embodied and affect-laden generalized worldviews that determine the construal of situations by different individuals. In other words, the same stimulus may be construed as an opportunity by some, or an obstacle by another – depending on the generalized worldview the individual has privately subscribed to and regardless of their own private personality-based inclinations. According to the authors, these universes foster social engagement due to perspectival solidarity achieved between agents. In their Europe-wide study concerning the effects of the economic and migration crises in Europe, the authors report the presence of 5 universes. The *Ordered* universe represents adherence to systems-based rules and regulations. The *Interpersonal Bond* universe represents valorization of social capital. The *Caring Society* universe is marked by a striving for community. *Niche of Belongingness* represents the effort to find a legitimate place in life for oneself and one’s loved ones. The *Others’ World* universe represents an attitude of helplessness and grit in the face of adversity.

### Social Axioms

Leung and Bond (2010) propose that human cognition is structured around generalized beliefs individuals hold about other people, social groups, institutions, society, and the spiritual realm. These general beliefs grant coherence in thinking about disparate elements in the world around us. Consequently, they are endorsed in an axiomatic fashion and serve to guide everyday conduct that involves the mutual presence of others. The authors distinguish between 5 social axioms, which they claim are universal in existence but variable in their manifestation: (a) *Religiosity*, representing an unquestioning attitude towards prescribed dogma; (b) *Social Complexity*, representing an attitude of strategic contingency based on interpersonal situations; (c) *Reward for Application*, representing a generalised attitude of striving for the betterment of one’s condition; (d) *Fate Control*, representing a generalised belief in the necessity to find a place for oneself in a powerful and controlled social order; and (e) *Social Cynicism*, representing a generalised belief that there is little one can do about the prevalent order to ameliorate one’s condition.

### Moral Foundations

Haidt (2012) claims that human beings are equipped with intuitive ethics that originate in their evolutionary survival. According to Haidt, these moral foundations provide us with flashes of affect in specific situations that serve to guide our conduct and shape our interpretation of the situation. Different cultures use these moral foundations differently to censure or otherwise particular action strategies. In the process of socialisation, individuals learn the normative rules that prevail around them. Violation of these rules triggers affective responses that predispose individuals towards determined responses. Haidt (2012) distinguished between 5 moral frameworks, that is (a) *Sanctity*, concerned with violations of degradation; (b) *Loyalty,* concerned with violation of trust and betrayal; (c) *Care*, concerned with violations resulting in harm; (d) *Fairness*, concerned with possibilities for cheating; and (e) *Authority*, concerned with subversion of a prevalent order. Haidt further proposes that additional frameworks may develop as a function of particular sociocultural concerns, such as the framework of *Liberty* in the US.

## The Psychological Gearbox: Mentalities, or is it Mindsets?

Clearly, a full theoretical rapprochement of these disparate research traditions is beyond the scope of the present contribution. However, the similarities reported in the descriptions of the underlying factors are palpable (see Table 1) and warrant consideration of the possibility of converging evidence.

**Table 1:** Five Factor Outputs in Generalized Beliefs Research Traditions

Symbolic Universes	Social Axioms	Moral Foundations
Ordered	Religiosity	Sanctity
Interpersonal Bond	Social Complexity	Loyalty
Caring Society	Reward for Application	Care
Niche of Belongingness	Fate Control	Fairness
Others’ World	Social Cynicism	Authority

All three research programmes have extended psychological inquiry beyond personality-based dispositions to generalized mental frames that mark individuals’ situated positioning relative to others on matters of social concern. The central idea investigated in these programmes is whether there are individual differences in how human beings interpret the events that happen to them and the world around them, controlling for personality structure. For instance, we can readily understand cultural differences in preferences for individual behavioural responses. These represent social norms of conduct. We can also readily understand how, within the same culture, different personality structures demonstrated by different individuals may precipitate different behavioural responses to the same stimulus. What we struggle to understand as readily is how the same individual, within the same culture, behaves in one way in one situation and a different way in another situation, when facing the same stimulus. We struggle with this because we expect the phenotype to match the genotype. Unless, that is, one considers a range of phenomena amenable to situational influences that also determine behaviour in part. I propose that *mentalities* are one such variable and that the three programmes considered above have tapped this very construct and similarly reported the same five-factor structure.

The notion of mentalities has been proposed by Burke (1986) to delineate a stress on collective attitudes that serve the purposes of practical reasoning and that are structured in terms of how people think as well as what they think. That is, mentalities represent *substantive cognition.* Etymologically, mentality refers to a ‘thinking style’. A related construct that arguably constitutes the other ground in this Rubin Vase is ‘mindset’, which etymologically represents a ‘thinking habit’. We have seen how mindsets extend the search for behavioural dispositions beyond the Big Five, as in the search for a Militant Extremist Mindset [MEM] cited above. I propose that mindsets are akin to specific mentalities and that this range of mentalities in human psychology constitutes the social-psychological mechanism by which individuals with pre-determined inclinations adapt their responses to ecological demands. To use a metaphor, mentalities are like a 5-piece psychological Swiss Army knife that enables individuals to psychologically adapt their mindset to tangibly situated environmental demands. If individual psychological adaptation to situational demands was not a successful strategy, then we would be justified in expecting personality structures to be much more enduring – you would have a knife for a knife job and a fork for a fork job. This is not how human behaviour manifests. To pose an example, G.I. Joe can go from being a foot soldier concerned with obliterating the enemy to a networking strategist coordinating logistics upon receiving a promotion. An adaptation in mindset might be called for, as one cannot adopt a combative stance in logistics. Many human beings routinely adapt to similar circumstances without issue. On the other hand, G. I. Joe may adapt by joining a security business at the end of his duty, where the same combative mindset may pay off in a novel environment. What this simple example makes clear is that neither personality structure nor sociocultural dimensions are very useful in understanding how an individual actuates their behavioural inclinations in situated circumstances.

## Conclusion

The replication crisis has precipitated renewed concerns about the nature of psychological phenomena and their manifestation in ecologically diverse contexts. Clearly, universalistic expectations concerning the manifestation of psychological phenomena are not supported. I have proposed that human psychology may demonstrate phenotype switching, which enables individuals to camouflage their inclinations for adaptation to situated ecological demands. This occurs by adopting a best-fitting mindset out of a range of mentalities that inhere in human psychology. I have argued that research looking at social dynamics suggests that human cognition is structured along five mentalities. I have then argued that this range of mentalities equips individuals with a multipurpose psychological mechanism to suit different ecological demands, like a Swiss Army knife provides a range of instruments to cater to different requirements.

Mentalities, therefore, provide human beings with the capacity for environmental calibration. Further research is required to determine what factors precipitate changes in mentality. Presumably, a mentality that is no longer functional due to situational constraints leads to maladaptive behaviour. This should precipitate psychological discomfort, or cognitive dissonance, that is reduced by revisiting the individual’s construal of the event, as per Festinger’s (1957) original findings. Phenotype switching of mentalities, therefore, enables individuals to adapt to changing circumstances.

I propose that mentalities work like a psychological gearbox for individuals to cope with their particular life terrain. The individual’s personality dispositions, partly rooted in genetics, are like a vehicle. Some vehicles are better than others, given particular environmental demands. A sports car may be a sight to behold at the racetrack, but in off-road conditions it will likely turn out to be a maladaptive choice. This is individual differences. But human beings demonstrate a capacity for adaptation in the same way that different cars can be driven in ways that accommodate ecological requirements – that is, environmental calibration. If driving a sports car off-road, one is advised to select a lower gear that permits the driver to ride slowly over bumps. If driving an off-road vehicle at the racetrack, one is advised to select a higher gear for the vehicle to increase and maintain velocity. Mentalities, therefore, are like gears – a range of five that enable the particular agent to calibrate their dispositional inclinations to ecological demands. Sometimes, it pays an individual to be open, positive, or innovative. This might be due to the fact that the individual finds themselves in a stable environment that provides for the individual’s needs. An open and positive mindset enables the individual to explore and reap opportunities for growth. At other times, it pays an individual to adopt a more cautious demeanour, to consider risk and worst-case scenarios. These help the individual overcome challenges and strife in times of adversity. Necessarily, for the behavioural response to be adaptive, the behavioural strategy needs to match the situated ecological demands. A growth mindset when facing bankruptcy is as maladaptive as a helpless mindset when opportunity beckons. Individuals, I argue, calibrate their behavioural strategies to environmental demands by adapting their psychological mindset to the situation, such that uncharacteristically cautious behaviour, for example, might start making sense to the bullish investor. Equally, uncharacteristically positive behaviour might start making sense to the re-settled refugee whose suspicious inclinations paid off in the past when facing a high-risk environment.

Clearly, adaptation in the human species is psychologically, not biologically, based – human beings do not demonstrate biological camouflage. Rather, in the human species, this involves an ability to change one’s mindset to a more profitable one. The capacity to switch mindsets is observable as situated cognition on the one hand, or relative stability on the other. Further research is required to determine the criteria that precipitate change; to determine whether the range of 5 mentalities is true to every individual or whether, like language, this might be a question of inherent potential contingent on socialisation; to determine whether psychological intervention may help individuals precipitate mentality changes; and to determine which mentalities might be indicated in specific circumstances. In any case, a focus on mentalities may provide answers concerning the potential for human adaptation. Changing one’s mentality or mindset involves the re-construal of particular events that opens up a profitable and cognitively consistent course of action – was this a good thing or a bad thing? Who gains, who loses? Should we act, or stay put? I conclude by suggesting that the study of psychological adaptation mechanisms may be key to understanding a host of dynamic behaviours such as creativity, decision-making, empathy, communication styles, resilience, and other behavioural strategies humans adopt to face the situated challenges they encounter over their lifetime.
